# Intractable Cough Associated With Renal Cell Carcinoma

**DOI:** 10.7759/cureus.18727

**Published:** 2021-10-12

**Authors:** Arminder Singh, Stephanie Everest, Lam Nguyen, Teshome Hailemichael

**Affiliations:** 1 Internal Medicine, Cape Fear Valley Medical Center, Fayetteville, USA; 2 School of Medicine, Campbell University School of Osteopathic Medicine, Lillington, USA

**Keywords:** nonproductive cough, cough, ace inhibitors, intractable cough, paraneoplastic syndromes, renal cell carcinoma

## Abstract

Renal cell carcinoma (RCC) is known to cause abdominal pain, hematuria, flank pain, fevers, night sweats, and weight loss, but its association with paraneoplastic syndromes such as intractable cough is rare. Here, we present the case of an 86-year-old female who presented with a persistent dry cough for two months. Computed tomography (CT) of the abdomen and pelvis with contrast revealed large left renal mass consistent with renal cell carcinoma spreading through Gerota’s fascia and metastatic to regional lymph nodes. Biopsy of the mass tested positive for renal cell carcinoma markers. Ultimately, the patient was deemed a nonsurgical candidate and treated with immunotherapy. In this case study, we discuss the rare but important clinical findings leading to the possible diagnosis of paraneoplastic cough secondary to RCC.

## Introduction

Renal cell carcinoma (RCC) is the most common primary renal neoplasm worldwide and the ninth most diagnosed cancer in the USA [1,2]. Few cases of RCC present as the “classic triad” of flank pain, hematuria, and flank mass, while others present with constitutional symptoms including fever, night sweats, and weight loss [3,4]. Widespread use of imaging modalities such as computed tomography (CT) and ultrasonography has aided in an increasing number of incidental RCC diagnoses. Interestingly, RCC has been associated with various paraneoplastic syndromes, including hypercalcemia, anemia, erythrocytosis, and Stauffer’s syndrome, and there have been some anecdotal reports of patients with RCC with intractable cough [4]. Here, we describe a possible case of intractable cough caused by paraneoplastic syndrome secondary to renal cell carcinoma. 

## Case presentation

An 86-year-old female with a history of atrial fibrillation, hypertension, hyperlipidemia, and status post pacemaker implantation presented to the emergency department (ED) with a persistent nonproductive cough for eight weeks and fever for three days. One month ago prior to presentation, her antihypertensive medication lisinopril was switched to losartan due to concern of an ace inhibitor-induced cough; however, her cough did not improve. CT of the abdomen and pelvis with contrast a month before the admission to the hospital had shown left-sided nephrolithiasis. Additionally, the patient reported decreased appetite, fatigue, and an eight-pound weight loss over the past month, and she endorsed left-sided abdominal pain, inguinal pain, and hematuria for three weeks. She denied dysuria, urinary frequency, back pain, chest pain, postnasal drip, heartburn, shortness of breath, or lower extremity edema. She denied any smoking in the past. She denied any recent exposure to animals or travel history. She denied any recent sick contact exposure. 

**Figure 1:**
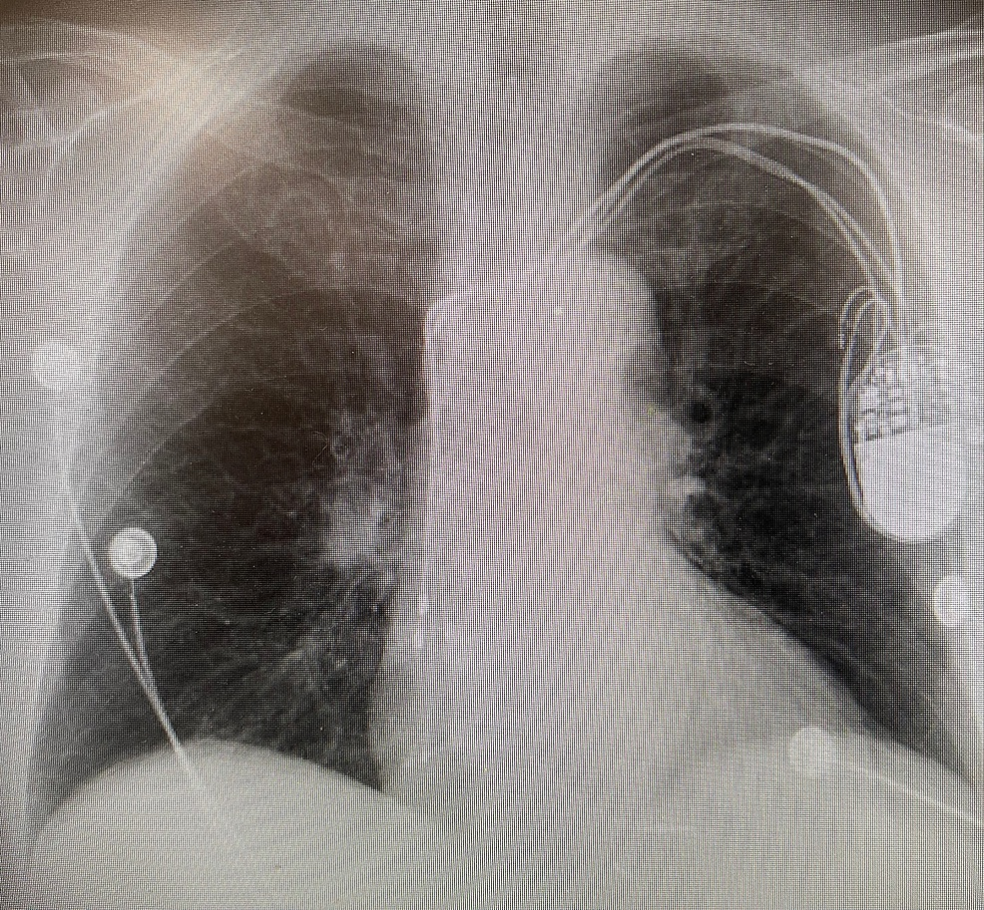
Chest radiograph demonstrating normal cardiomediastinal contours, pacemaker, no airspace consolidation, no pleural effusion, no pneumothorax, and no acute bony abnormalities

**Figure 2:**
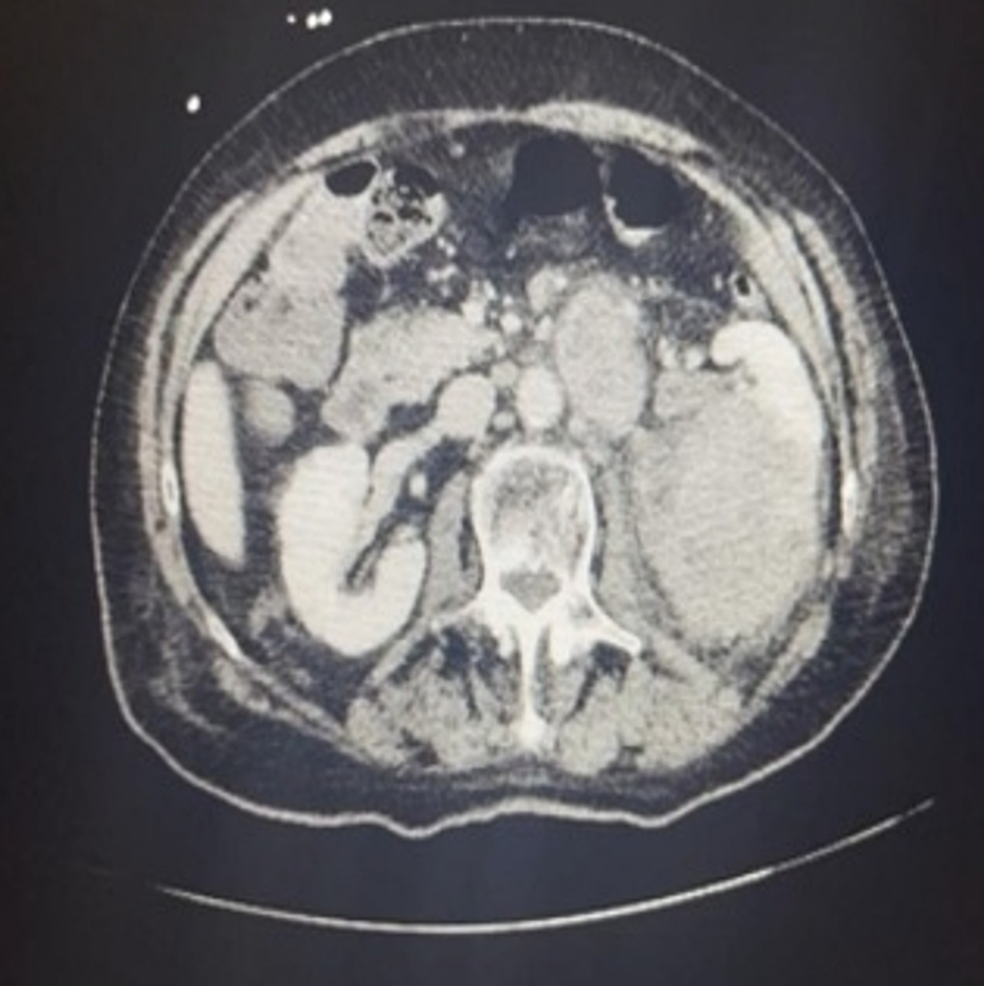
Computed tomography of the large heterogeneous solid renal mass on the left measuring 9.7 x 7.8 x 8.6 cm with extension through Gerota’s fascia and metastases to regional lymph nodes

**Figure 3:**
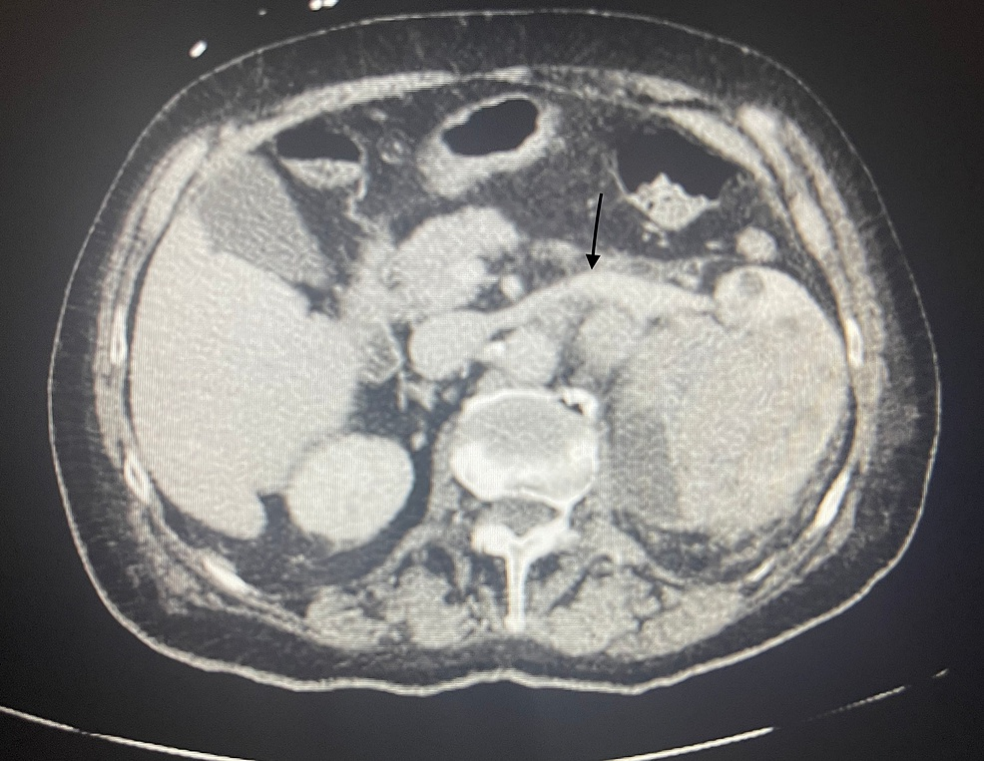
Computed tomography demonstrating the large heterogeneous solid renal mass on the left measuring 9.7 x 7.8 x 8.6 cm with extension through Gerota’s fascia and metastases to regional lymph nodes with no renal vein involvement (arrow) or distant metastatic foci noted

Initial vitals showed a blood pressure of 143/88 mmHg, heart rate of 115 beats/minute, temperature of 39.4°C (102.9°F), respiratory rate of 16 breaths/minute, and oxygen saturation of 91% on room air. Oxygen saturation improved to 99% on room air within an hour of presentation to ED. Physical examination showed tachycardia, dry cough, and mild left lower abdominal tenderness to palpation. Laboratory test results were significant for leukocytosis, mild anemia, and an elevated ESR and CRP. Urinalysis was positive with 3+ leukocyte esterase, 32 WBCs, 3+ blood, >180 RBCs, and 1+ bacteria. A PCR test for SARS-CoV-2 was negative. Blood culture drawn from two different peripheral sites resulted in no growth at 120 hours. Lactic acid was within the normal range at 1.5 mmol/L. Procalcitonin was within the normal range at 0.13 ng/mL. A chest radiograph demonstrated normal cardiomediastinal contours, pacemaker, no airspace consolidation, no pleural effusion, no pneumothorax, and no acute bony abnormalities (Figure 1). CT of the abdomen and pelvis with contrast revealed a large heterogeneous solid renal mass on the left measuring 9.7 x 7.8 x 8.6 cm that extended through Gerota’s fascia with metastases to regional lymph nodes (Figure 2). On CT, there was no evidence of renal vein involvement or distant metastatic foci (Figure 3). CT-guided needle biopsy of the kidney was then performed to make the final diagnosis. Pathology showed that the tumor was consistent with renal cell carcinoma based on the immunoprofile of positive epithelial markers of AE1/AE3, CK19, and CK7 and positive PAX8, CD10, RCC, and vimentin immunostains. The patient was deemed unfit for surgical intervention due to her age and advanced stage of cancer. Thus, she was advised to be discharged and followed up with outpatient oncology for palliative medical treatment of immunotherapy and tyrosine kinase inhibitors. Of note, the chest X-ray and lung lower lobes on abdominal CT results indicated that pulmonary metastases were very unlikely. 

 

## Discussion

The clinical triad of flank pain, palpable flank mass, and hematuria has been historically used to diagnose RCC. Also, patients with RCC often present with fever, fatigue, sweats, weight loss, neutrophilia, eosinophilia, thrombocytosis, or anemia [3,4]. The widespread availability and usage of diagnostic tools such as CT and ultrasound have helped make the diagnosis of RCC earlier, and the clinical triad has become less frequent [4]. Moreover, paraneoplastic symptoms such as hypertension, hypercalcemia, and polycythemia have been observed in patients with RCC [4,5]. Chronic intractable cough has also been recorded in RCC, and its etiology is mostly associated with pulmonary metastases or tumor mass-induced diaphragmatic irritation [6]. However, there have been rare reports of persistent cough in patients with RCC without metastatic lung tumors [7]. Interestingly, the coughs in these cases resolved after the complete removal of the cancer via nephrectomy, demonstrating that the symptom was a paraneoplastic effect of RCC. Nephrectomy proved to be a definitive treatment for these patients [8].

In our patient, the negative chest X-ray and CT of the abdomen and pelvis with a view of the lower lobes demonstrated that pulmonary metastases were unlikely to be the etiology of her cough. It is hard to be certain about pulmonary metastasis in this patient given that no CT of the chest was performed for the patient. The abdominal CT showed that there was no approximation between the patient's diaphragm and her renal tumor, which ruled out mass-induced diaphragmatic irritation as an etiology. ACE-induced cough was also unlikely in the patient due to her cough persisting after lisinopril discontinuation. There were no other obvious triggers causing the cough symptom. Given the chest X-ray revealed no consolidation or infiltration and procalcitonin was negative, pneumonia was unlikely in this patient. Gastroesophageal reflux disease (GERD) is less likely the reason for the cough due to the patient denying heartburn. The cough is not likely due to a postnasal drip as she reported no postnasal symptoms. She has no history of smoking, making smoking less of an irritant for the cough, and no past medical history of COPD or asthma, making her cough less suggestive of COPD and asthma exacerbation. Hence, RCC was thought to be the cause of her symptoms. The exact pathophysiology of cough associated with RCC has not yet been established. Due to the lack of travel history and exposure to animals, her cough is unlikely due to environmental triggers. However, there have been several hypotheses that suggested that the cough is caused by prostaglandins, cytokines, and bradykinin secreted from cancer cells [5,7]. Interleukin-6 (IL-6) levels have been shown to be elevated in patients with RCC, and its inflammatory effects have been a proposed mechanism for multiple paraneoplastic symptoms in these patients [9]. A drop in IL-6 levels has also been reported following tumor removal and associated with the resolution of cough in patients with RCC [8]. Unfortunately, IL-6 levels were not measured in our patient, and further research is necessary to understand the role of IL-6 in the medical treatment of RCC. As previously discussed, nephrectomy has been reported to immediately resolve or improve paraneoplastic cough symptoms; thus, surgery may serve as the mainstay treatment for this patient population subset. Our patient was not a candidate for nephrectomy and was started on chemotherapy with cabozantinib and nivolumab every two weeks.

## Conclusions

Extrapulmonary etiologies should be considered in the diagnostic workup of those presenting with a cough. In this report, we discussed the possible paraneoplastic effects of renal cell carcinoma, which provoked an intractable cough. Cough is a very common symptom and can be caused by many etiologies. In this case, cough can be contributed to RCC given common etiologies, such as GERD, pneumonia, COPD, asthma, postnasal drip, COVID-19, smoking, hypersensitivity to animals or environmental triggers, and ACE inhibitor-induced cough, were ruled out. While findings of renal cell carcinoma are typically incidental, we propose that clinicians evaluate for renal cell carcinoma in patients with a persistent cough despite normal findings on chest imaging. Future research should focus on further defining the pathophysiology of RCC-related paraneoplastic syndromes to better understand symptom-based treatment for nonsurgical candidates such as the patient presented in this case. 
